# Supplementary material for: Plasma Proteomic Analysis in Morquio A Disease
Source: Int J Mol Sci. 2021 Jun 7;22(11):6165. doi: 10.3390/ijms22116165 (PMC8201332; doi:10.3390/ijms22116165)
Supplement: Supplementary file 1 [file ijms-22-06165-s001.zip › ijms-1234068-supplementary.pdf]

Table S1. Number of proteins identified per sample and number of proteins identified in all or all but one samples per group. Only proteins with a false discovery rate <1% were selected.

**Patients and CG**	**Sample ID**	**Proteins Identified per Sample (*n*)**	**Proteins Identified in all or all but one samples (*n*)**
UG	UG 1	268	113
	UG 2	215	
	UG 3	158	
	UG 4	173	
	UG 5	187	
	UG 6	172	
	UG 7	176	
	UG 8	204	
ERT-a Group	ERT-a 1	257	161
	ERT-a 2	166	
	ERT-a 3	181	
	ERT-a 4	153	
	ERT-a 5	186	
ERT-b Group	ERT-b 1	228	130
	ERT-b 2	128	
	ERT-b 3	128	
	ERT-b 4	122	
	ERT-b 5	175	
CG	CG 1	226	164
	CG 2	182	
	CG 3	207	
	CG 4	211	
	CG 5	225	
	CG6	239	

Abbreviations: CG, control group; MPS IVA, mucopolysaccharidosis type IVA; ERT-a, MPS IVA patients sampled before ERT; ERT-b, MPS IVA patients sampled 24 h after ERT; UG, untreated group.

Table S2. List of proteins used for the qualitative study of proteins.

Code Uniprot	Protein UG
P02656	Apolipoprotein C-III
Q06033	Inter-alpha-trypsin inhibitor heavy chain H3
P00918	Carbonic anhydrase 2
P02675	Fibrinogen beta chain
P01594	Immunoglobulin kappa variable 1-33
P01042	Kininogen-1
P05155	lasma protease C1 inhibitor
P01019	Angiotensinogen
P19823	Inter-alpha-trypsin inhibitor heavy chain H2
P01859	Immunoglobulin heavy constant gamma 2
P01860	Immunoglobulin heavy constant gamma 3
P04264	Keratin, type II cytoskeletal 1
P02774	Vitamin D-binding protein
P00450	Ceruloplasmin
P80748	Immunoglobulin lambda variable 3-21
P01008	Antithrombin-III
P02751	Fibronectin
P02647	Apolipoprotein A-I
P06681	Complement C2
P06310	Immunoglobulin kappa variable 2-30
P06276	Cholinesterase
P32119	Peroxiredoxin-2
A0A0B4J1V0	Immunoglobulin heavy variable 3-15
P02649	Apolipoprotein E
Q96KN2	Beta-Ala-His dipeptidase
P01876	Immunoglobulin heavy constant alpha 1
P02745	Complement C1q subcomponent subunit A
A0A0A0MS15	Immunoglobulin heavy variable 3-49
P01023	Alpha-2-macroglobulin
P02671	Fibrinogen alpha chain
A0A0B4J1U3	Immunoglobulin lambda variable 1-36
P07225	Vitamin K-dependent protein S
P63261	Actin, cytoplasmic 2
P01871	Immunoglobulin heavy constant mu
P43652	Afamin
P05452	Tetranectin
P01780	Immunoglobulin heavy variable 3-7
P29622	Kallistatin
P51884	Lumican
P05543	Thyroxine-binding globulin
P02766	Transthyretin
Q15848	Adiponectin
A0A0C4DH31	Immunoglobulin heavy variable 1-18
P08603	Complement factor H

P01011	Alpha-1-antichymotrypsin
P00751	Complement factor B
P0C0L4	Complement C4-A
P04180	Phosphatidylcholine-sterol acyltransferase
P36955	Pigment epithelium-derived factor
P01602	Immunoglobulin kappa variable 1-5
A0A0B4J1U7	Immunoglobulin heavy variable 6-1
P43251	Biotinidase
O14791	Apolipoprotein L1
P0C0L5	Complement C4-B
P01877	Immunoglobulin heavy constant alpha 2
P06727	Apolipoprotein A-IV
P02042	Hemoglobin subunit delta
P02750	Leucine-rich alpha-2-glycoprotein
P01834	Immunoglobulin kappa constant
Q08380	Galectin-3-binding protein
P02652	Apolipoprotein A-II
P02765	Alpha-2-HS-glycoprotein
Q9UK55	Protein Z-dependent protease inhibitor
P01024	Complement C3
A0A0B4J1X5	Immunoglobulin heavy variable 3-74
P05090	Apolipoprotein D
O95445	Apolipoprotein M
P01615	Immunoglobulin kappa variable 2D-28
P13645	Keratin, type I cytoskeletal 10
P08185	Corticosteroid-binding globulin
P01701	Immunoglobulin lambda variable 1-51
P01857	Immunoglobulin heavy constant gamma 1
P27169	Serum paraoxonase/arylesterase 1
A0A0C4DH38	Immunoglobulin heavy variable 5-51
P05154	Plasma serine protease inhibitor
P04217	Alpha-1B-glycoprotein
P04004	Vitronectin
B9A064	Immunoglobulin lambda-like polypeptide 5
P01611	Immunoglobulin kappa variable 1D-12
P19827	Inter-alpha-trypsin inhibitor heavy chain H1
P02747	Complement C1q subcomponent subunit C
P01619	Immunoglobulin kappa variable 3-20
P06312	Immunoglobulin kappa variable 4-1
P04211	Immunoglobulin lambda variable 7-43
P68871	Hemoglobin subunit beta
P01706	Immunoglobulin lambda variable 2-11
P02743	Serum amyloid P-component
P02790	Hemopexin
A0A0C4DH24	Immunoglobulin kappa variable 6-21
P01700	Immunoglobulin lambda variable 1-47

Q96PD5	N-acetylmuramoyl-L-alanine amidase
P00738	Haptoglobin
P01591	Immunoglobulin J chain
P02654	Apolipoprotein C-I
P01601	Immunoglobulin kappa variable 1D-16
P02655	Apolipoprotein C-II
P06396	Gelsolin
P25311	Zinc-alpha-2-glycoprotein
P02787	Serotransferrin
P06702	Protein S100-A9
P35858	Insulin-like growth factor-binding protein complex acid labile subunit
P10909	Clusterin
P01721	Immunoglobulin lambda variable 6-57
P08697	Alpha-2-antiplasmin
P04433	Immunoglobulin kappa variable 3-11
P0DP03	Immunoglobulin heavy variable 3-30-5
P22792	Carboxypeptidase N subunit 2
P02768	Albumin
Q03591	Complement factor H-related protein 1
A0A0B4J1V2	Immunoglobulin heavy variable 2-26
Q14624	Inter-alpha-trypsin inhibitor heavy chain H4
A0A0C4DH68	Immunoglobulin kappa variable 2-24
A0A0B4J1Y9	Immunoglobulin heavy variable 3-72
P01009	Alpha-1-antitrypsin
P02679	Fibrinogen gamma chain
P02753	Retinol-binding protein 4
P02746	Complement C1q subcomponent subunit B
P02763	Alpha-1-acid glycoprotein 1
P05156	Complement factor I
P01861	Immunoglobulin heavy constant gamma 4
P35542	Serum amyloid A-4 protein
P49908	Selenoprotein P
P01624	Immunoglobulin kappa variable 3-15
P01031	Complement C5
P04430	Immunoglobulin kappa variable 1-16
P02749	Beta-2-glycoprotein 1
A0A075B6I0	Immunoglobulin lambda variable 8-61
Q96IY4	Carboxypeptidase B2
P19652	Alpha-1-acid glycoprotein 2
P00915	Carbonic anhydrase 1
P02760	Protein AMBP
P69905	Hemoglobin subunit alpha
P05546	Heparin cofactor 2
P04114	Apolipoprotein B-100
P35527	Keratin, type I cytoskeletal 9
P00739	Haptoglobin-related protein

P00734	Prothrombin
P04196	Histidine-rich glycoprotein
P01743	Immunoglobulin heavy variable 1-46
A0A0A0MT36	Immunoglobulin kappa variable 6D-21
A0A0J9YX35	Immunoglobulin heavy variable 3-64D
A0A0C4DH34	Immunoglobulin heavy variable 4-28
P00488	Coagulation factor XIII A chain
A0A075B6J9	Immunoglobulin lambda variable 2-18
P18428	Lipopolysaccharide-binding protein
P04432	Immunoglobulin kappa variable 1D-39
A0A0C4DH25	Immunoglobulin kappa variable 3D-20
O00391	Sulfhydryl oxidase 1
P01599	Immunoglobulin kappa variable 1-17
P13796	Plastin-2
O43866	CD5 antigen-like
P22352	Glutathione peroxidase 3
O75882	Attractin
P01714	Immunoglobulin lambda variable 3-19
P01709	Immunoglobulin lambda variable 2-8
P15814	Immunoglobulin lambda-like polypeptide 1
P01717	Immunoglobulin lambda variable 3-25
A0A0B4J1Y8	Immunoglobulin lambda variable 9-49
P00742	Coagulation factor X
P55058	Phospholipid transfer protein
P05109	Protein S100-A8
P04278	Sex hormone-binding globulin
P15169	Carboxypeptidase N catalytic chain
P11597	Cholesteryl ester transfer protein

Code Uniprot	ERT-a
P02656	Apolipoprotein C-III
Q06033	Inter-alpha-trypsin inhibitor heavy chain H3
P02675	Fibrinogen beta chain
P01594	Immunoglobulin kappa variable 1-33
P01042	Kininogen-1
P05155	lasma protease C1 inhibitor
P01019	Angiotensinogen
P19823	Inter-alpha-trypsin inhibitor heavy chain H2
P01859	Immunoglobulin heavy constant gamma 2
P04264	Keratin, type II cytoskeletal 1
P02774	Vitamin D-binding protein
P00450	Ceruloplasmin
P01008	Antithrombin-III
P02647	Apolipoprotein A-I
P06681	Complement C2
P06310	Immunoglobulin kappa variable 2-30
P06276	Cholinesterase

P02649	Apolipoprotein E
P01876	Immunoglobulin heavy constant alpha 1
P01023	Alpha-2-macroglobulin
P02671	Fibrinogen alpha chain
P07225	Vitamin K-dependent protein S
P01871	Immunoglobulin heavy constant mu
P63261	Actin, cytoplasmic 2
P43652	Afamin
P35908	Keratin, type II cytoskeletal 2 epidermal
P29622	Kallistatin
P51884	Lumican
P05543	Thyroxine-binding globulin
P02766	Transthyretin
P08603	Complement factor H
P01011	Alpha-1-antichymotrypsin
P00751	Complement factor B
P12259	Coagulation factor V
P0C0L4	Complement C4-A
P36955	Pigment epithelium-derived factor
P43251	Biotinidase
Q9NZP8	Complement C1r subcomponent-like protein
O14791	Apolipoprotein L1
P06727	Apolipoprotein A-IV
P02042	Hemoglobin subunit delta
P02750	Leucine-rich alpha-2-glycoprotein
A0A075B6H9	Immunoglobulin lambda variable 4-69
P01834	Immunoglobulin kappa constant
Q08380	Galectin-3-binding protein
P02652	Apolipoprotein A-II
P02765	Alpha-2-HS-glycoprotein
Q9UK55	Protein Z-dependent protease inhibitor
P01024	Complement C3
P05090	Apolipoprotein D
O95445	Apolipoprotein M
P01615	Immunoglobulin kappa variable 2D-28
P13645	Keratin, type I cytoskeletal 10
P08185	Corticosteroid-binding globulin
P01857	Immunoglobulin heavy constant gamma 1
P27169	Serum paraoxonase/arylesterase 1
P15169	Carboxypeptidase N catalytic chain
P05154	Plasma serine protease inhibitor
P04217	Alpha-1B-glycoprotein
B9A064	Immunoglobulin lambda-like polypeptide 5
P11597	Cholesteryl ester transfer protein
P19827	Inter-alpha-trypsin inhibitor heavy chain H1
A0A0B4J1Y8	Immunoglobulin lambda variable 9-49

P01619	Immunoglobulin kappa variable 3-20
P68871	Hemoglobin subunit beta
P02743	Serum amyloid P-component
P02790	Hemopexin
P01700	Immunoglobulin lambda variable 1-47
Q96PD5	N-acetylmuramoyl-L-alanine amidase
P00738	Haptoglobin
P01591	Immunoglobulin J chain
P02654	Apolipoprotein C-I
P25311	Zinc-alpha-2-glycoprotein
P06396	Gelsolin
P02787	Serotransferrin
P35858	Insulin-like growth factor-binding protein complex acid labile subunit
P10909	Clusterin
P08697	Alpha-2-antiplasmin
P55058	Phospholipid transfer protein
P22792	Carboxypeptidase N subunit 2
P02768	Albumin
Q14624	Inter-alpha-trypsin inhibitor heavy chain H4
P01009	Alpha-1-antitrypsin
P02679	Fibrinogen gamma chain
P02753	Retinol-binding protein 4
P0DOY3	Immunoglobulin lambda constant 3
P35542	Serum amyloid A-4 protein
P01031	Complement C5
P02749	Beta-2-glycoprotein 1
A0A075B6I0	Immunoglobulin lambda variable 8-61
P19652	Alpha-1-acid glycoprotein 2
P00915	Carbonic anhydrase 1
P02760	Protein AMBP
P69905	Hemoglobin subunit alpha
P05546	Heparin cofactor 2
P04114	Apolipoprotein B-100
P04196	Histidine-rich glycoprotein
P04278	Sex hormone-binding globulin
O00391	Sulfhydryl oxidase 1
A0A0B4J1U7	Immunoglobulin heavy variable 6-1
A0M8Q6	Immunoglobulin lambda constant 7
A0A0C4DH68	Immunoglobulin kappa variable 2-24
P35527	Keratin, type I cytoskeletal 9
P01880	Immunoglobulin heavy constant delta
P04040	Catalase
P01714	Immunoglobulin lambda variable 3-19
P05452	Tetranectin
P04180	Phosphatidylcholine-sterol acyltransferase
P01602	Immunoglobulin kappa variable 1-5

P0C0L5	Complement C4-B
P01721	Immunoglobulin lambda variable 6-57
P02763	Alpha-1-acid glycoprotein 1
P01861	Immunoglobulin heavy constant gamma 4
Q96IY4	Carboxypeptidase B2
P00734	Prothrombin
O75882	Attractin
P00488	Coagulation factor XIII A chain
P02747	Complement C1q subcomponent subunit C
P04211	Immunoglobulin lambda variable 7-43
P02746	Complement C1q subcomponent subunit B
P32119	Peroxiredoxin-2
A0A0B4J1V0	Immunoglobulin heavy variable 3-15
Q15848	Adiponectin
P02655	Apolipoprotein C-II
P02751	Fibronectin
P04003	C4b-binding protein alpha chain
P0DP08	Immunoglobulin heavy variable 4-38-2
A0A0C4DH34	Immunoglobulin heavy variable 4-28
P80108	Phosphatidylinositol-glycan-specific phospholipase D
Code Uniprot	ERT-b
Q06033	Inter-alpha-trypsin inhibitor heavy chain H3
P02675	Fibrinogen beta chain
P01594	Immunoglobulin kappa variable 1-33
P01042	Kininogen-1
P05155	Plasma protease C1 inhibitor
P01019	Angiotensinogen
P19823	Inter-alpha-trypsin inhibitor heavy chain H2
P01859	Immunoglobulin heavy constant gamma 2
P04264	Keratin, type II cytoskeletal 1
P00450	Ceruloplasmin
P01008	Antithrombin-III
P02647	Apolipoprotein A-I
P06681	Complement C2
P06310	Immunoglobulin kappa variable 2-30
P06276	Cholinesterase
P02649	Apolipoprotein E
P01876	Immunoglobulin heavy constant alpha 1
P01023	Alpha-2-macroglobulin
P02671	Fibrinogen alpha chain
A0A0C4DH25	Immunoglobulin kappa variable 3D-20
P07225	Vitamin K-dependent protein S
P01871	Immunoglobulin heavy constant mu
P35908	Keratin, type II cytoskeletal 2 epidermal
P29622	Kallistatin
P51884	Lumican

P05543	Thyroxine-binding globulin
P02766	Transthyretin
P01011	Alpha-1-antichymotrypsin
P00751	Complement factor B
P0C0L4	Complement C4-A
P36955	Pigment epithelium-derived factor
P04003	C4b-binding protein alpha chain
Q9NZP8	Complement C1r subcomponent-like protein
O14791	Apolipoprotein L1
P06727	Apolipoprotein A-IV
P02750	Leucine-rich alpha-2-glycoprotein
P01834	Immunoglobulin kappa constant
Q08380	Galectin-3-binding protein
P02652	Apolipoprotein A-II
P02765	Alpha-2-HS-glycoprotein
Q9UK55	Protein Z-dependent protease inhibitor
P01024	Complement C3
P05090	Apolipoprotein D
P0DP08	Immunoglobulin heavy variable 4-38-2
P01615	Immunoglobulin kappa variable 2D-28
P13645	Keratin, type I cytoskeletal 10
P08185	Corticosteroid-binding globulin
P01857	Immunoglobulin heavy constant gamma 1
P27169	Serum paraoxonase/arylesterase 1
P15169	Carboxypeptidase N catalytic chain
P05154	Plasma serine protease inhibitor
P04217	Alpha-1B-glycoprotein
B9A064	Immunoglobulin lambda-like polypeptide 5
P11597	Cholesteryl ester transfer protein
P19827	Inter-alpha-trypsin inhibitor heavy chain H1
A0A0B4J1Y8	Immunoglobulin lambda variable 9-49
P68871	Hemoglobin subunit beta
P02790	Hemopexin
P01700	Immunoglobulin lambda variable 1-47
Q96PD5	N-acetylmuramoyl-L-alanine amidase
P00738	Haptoglobin
P01591	Immunoglobulin J chain
P25311	Zinc-alpha-2-glycoprotein
P06396	Gelsolin
P02787	Serotransferrin
P35858	Insulin-like growth factor-binding protein complex acid labile subunit
P10909	Clusterin
P08697	Alpha-2-antiplasmin
P55058	Phospholipid transfer protein
P22792	Carboxypeptidase N subunit 2
P02768	Albumin

Q14624	Inter-alpha-trypsin inhibitor heavy chain H4
P01009	Alpha-1-antitrypsin
P02679	Fibrinogen gamma chain
P02753	Retinol-binding protein 4
P0DOY3	Immunoglobulin lambda constant 3
P01861	Immunoglobulin heavy constant gamma 4
P01031	Complement C5
P19652	Alpha-1-acid glycoprotein 2
P02760	Protein AMBP
P69905	Hemoglobin subunit alpha
P05546	Heparin cofactor 2
P04114	Apolipoprotein B-100
P35527	Keratin, type I cytoskeletal 9
P00734	Prothrombin
P04196	Histidine-rich glycoprotein
P43652	Afamin
P08603	Complement factor H
P02751	Fibronectin
O00391	Sulfhydryl oxidase 1
A0A0C4DH38	Immunoglobulin heavy variable 5-51
P00488	Coagulation factor XIII A chain
P01721	Immunoglobulin lambda variable 6-57
P02746	Complement C1q subcomponent subunit B
P02763	Alpha-1-acid glycoprotein 1
P01880	Immunoglobulin heavy constant delta
P63261	Actin, cytoplasmic 2
P12259	Coagulation factor V
P04180	Phosphatidylcholine-sterol acyltransferase
A0A075B6I0	Immunoglobulin lambda variable 8-61
P02774	Vitamin D-binding protein
Q96KN2	Beta-Ala-His dipeptidase
P43251	Biotinidase
P01877	Immunoglobulin heavy constant alpha 2
A0A075B6H9	Immunoglobulin lambda variable 4-69
A0A0C4DH34	Immunoglobulin heavy variable 4-28
P02747	Complement C1q subcomponent subunit C
A0M8Q6	Immunoglobulin lambda constant 7
P35542	Serum amyloid A-4 protein
P02749	Beta-2-glycoprotein 1
P80748	Immunoglobulin lambda variable 3-21
P01602	Immunoglobulin kappa variable 1-5
Code Uniprot	CG
P02656	Apolipoprotein C-III
Q06033	Inter-alpha-trypsin inhibitor heavy chain H3
P00918	Carbonic anhydrase 2
P02675	Fibrinogen beta chain

P01594	Immunoglobulin kappa variable 1-33
P01042	Kininogen-1
P05155	Plasma protease C1 inhibitor
P01019	Angiotensinogen
P19823	Inter-alpha-trypsin inhibitor heavy chain H2
P01859	Immunoglobulin heavy constant gamma 2
P01860	Immunoglobulin heavy constant gamma 3
P04264	Keratin, type II cytoskeletal 1
P02774	Vitamin D-binding protein
P00450	Ceruloplasmin
P80748	Immunoglobulin lambda variable 3-21
P01008	Antithrombin-III
P02751	Fibronectin
P02647	Apolipoprotein A-I
P06681	Complement C2
P06310	Immunoglobulin kappa variable 2-30
P06276	Cholinesterase
P32119	Peroxiredoxin-2
A0A0B4J1V0	Immunoglobulin heavy variable 3-15
P02649	Apolipoprotein E
Q96KN2	Beta-Ala-His dipeptidase
P01876	Immunoglobulin heavy constant alpha 1
P02745	Complement C1q subcomponent subunit A
A0A0A0MS15	Immunoglobulin heavy variable 3-49
P01023	Alpha-2-macroglobulin
P02671	Fibrinogen alpha chain
A0A0B4J1U3	Immunoglobulin lambda variable 1-36
P07225	Vitamin K-dependent protein S
P63261	Actin, cytoplasmic 2
P01871	Immunoglobulin heavy constant mu
P43652	Afamin
P05452	Tetranectin
P01780	Immunoglobulin heavy variable 3-7
P29622	Kallistatin
P51884	Lumican
P05543	Thyroxine-binding globulin
P02766	Transthyretin
Q15848	Adiponectin
A0A0C4DH31	Immunoglobulin heavy variable 1-18
P08603	Complement factor H
P01011	Alpha-1-antichymotrypsin
P00751	Complement factor B
P0C0L4	Complement C4-A
P04180	Phosphatidylcholine-sterol acyltransferase
P36955	Pigment epithelium-derived factor
P01602	Immunoglobulin kappa variable 1-5

A0A0B4J1U7	Immunoglobulin heavy variable 6-1
P43251	Biotinidase
O14791	Apolipoprotein L1
P0C0L5	Complement C4-B
P01877	Immunoglobulin heavy constant alpha 2
P06727	Apolipoprotein A-IV
P02042	Hemoglobin subunit delta
P02750	Leucine-rich alpha-2-glycoprotein
P01834	Immunoglobulin kappa constant
Q08380	Galectin-3-binding protein
P02652	Apolipoprotein A-II
P02765	Alpha-2-HS-glycoprotein
Q9UK55	Protein Z-dependent protease inhibitor
P01024	Complement C3
A0A0B4J1X5	Immunoglobulin heavy variable 3-74
P05090	Apolipoprotein D
O95445	Apolipoprotein M
P01615	Immunoglobulin kappa variable 2D-28
P13645	Keratin, type I cytoskeletal 10
P08185	Corticosteroid-binding globulin
P01701	Immunoglobulin lambda variable 1-51
P01857	Immunoglobulin heavy constant gamma 1
P27169	Serum paraoxonase/arylesterase 1
A0A0C4DH38	Immunoglobulin heavy variable 5-51
P05154	Plasma serine protease inhibitor
P04217	Alpha-1B-glycoprotein
P04004	Vitronectin
B9A064	Immunoglobulin lambda-like polypeptide 5
P01611	Immunoglobulin kappa variable 1D-12
P19827	Inter-alpha-trypsin inhibitor heavy chain H1
P02747	Complement C1q subcomponent subunit C
P01619	Immunoglobulin kappa variable 3-20
P06312	Immunoglobulin kappa variable 4-1
P04211	Immunoglobulin lambda variable 7-43
P68871	Hemoglobin subunit beta
P01706	Immunoglobulin lambda variable 2-11
P02743	Serum amyloid P-component
P02790	Hemopexin
A0A0C4DH24	Immunoglobulin kappa variable 6-21
P01700	Immunoglobulin lambda variable 1-47
Q96PD5	N-acetylmuramoyl-L-alanine amidase
P00738	Haptoglobin
P01591	Immunoglobulin J chain
P02654	Apolipoprotein C-I
P01601	Immunoglobulin kappa variable 1D-16
P02655	Apolipoprotein C-II

P06396	Gelsolin
P25311	Zinc-alpha-2-glycoprotein
P02787	Serotransferrin
P06702	Protein S100-A9
P35858	Insulin-like growth factor-binding protein complex acid labile subunit
P10909	Clusterin
P01721	Immunoglobulin lambda variable 6-57
P08697	Alpha-2-antiplasmin
P04433	Immunoglobulin kappa variable 3-11
P0DP03	Immunoglobulin heavy variable 3-30-5
P22792	Carboxypeptidase N subunit 2
P02768	Albumin
Q03591	Complement factor H-related protein 1
A0A0B4J1V2	Immunoglobulin heavy variable 2-26
Q14624	Inter-alpha-trypsin inhibitor heavy chain H4
A0A0C4DH68	Immunoglobulin kappa variable 2-24
A0A0B4J1Y9	Immunoglobulin heavy variable 3-72
P01009	Alpha-1-antitrypsin
P02679	Fibrinogen gamma chain
P02753	Retinol-binding protein 4
P02746	Complement C1q subcomponent subunit B
P02763	Alpha-1-acid glycoprotein 1
P05156	Complement factor I
P01861	Immunoglobulin heavy constant gamma 4
P35542	Serum amyloid A-4 protein
P49908	Selenoprotein P
P01624	Immunoglobulin kappa variable 3-15
P01031	Complement C5
P04430	Immunoglobulin kappa variable 1-16
P02749	Beta-2-glycoprotein 1
A0A075B6I0	Immunoglobulin lambda variable 8-61
Q96IY4	Carboxypeptidase B2
P19652	Alpha-1-acid glycoprotein 2
P00915	Carbonic anhydrase 1
P02760	Protein AMBP
P69905	Hemoglobin subunit alpha
P05546	Heparin cofactor 2
P04114	Apolipoprotein B-100
P35527	Keratin, type I cytoskeletal 9
P00739	Haptoglobin-related protein
P00734	Prothrombin
P04196	Histidine-rich glycoprotein
P01743	Immunoglobulin heavy variable 1-46
A0A0A0MT36	Immunoglobulin kappa variable 6D-21
A0A0J9YX35	Immunoglobulin heavy variable 3-64D
A0A0C4DH34	Immunoglobulin heavy variable 4-28

P00488	Coagulation factor XIII A chain
A0A075B6J9	Immunoglobulin lambda variable 2-18
P18428	Lipopolysaccharide-binding protein
P04432	Immunoglobulin kappa variable 1D-39
A0A0C4DH25	Immunoglobulin kappa variable 3D-20
O00391	Sulfhydryl oxidase 1
P01599	Immunoglobulin kappa variable 1-17
P13796	Plastin-2
O43866	CD5 antigen-like
P22352	Glutathione peroxidase 3
O75882	Attractin
P01714	Immunoglobulin lambda variable 3-19
P01709	Immunoglobulin lambda variable 2-8
P15814	Immunoglobulin lambda-like polypeptide 1
P01717	Immunoglobulin lambda variable 3-25
A0A0B4J1Y8	Immunoglobulin lambda variable 9-49
P00742	Coagulation factor X
P55058	Phospholipid transfer protein
P05109	Protein S100-A8
P04278	Sex hormone-binding globulin
P15169	Carboxypeptidase N catalytic chain
P11597	Cholesteryl ester transfer protein
